# Outcomes and Safety of Same-Day Discharge Following Minimally Invasive Abdominal Surgery: A Systematic Review

**DOI:** 10.7759/cureus.104349

**Published:** 2026-02-26

**Authors:** Cristian Israel Sarmiento Bonilla, Andrés Sebastián Estrella López, María Alejandra Lima Velasquez, Sara Marianne García Escribá, Marcelo Adolfo Perez Benito

**Affiliations:** 1 Surgery, Hospital de Especialidades, Unidad Médica de Alta Especialidad (UMAE) No. 71, Torreón, MEX; 2 Public Health, NeoScientia Consulting Group, Quito, ECU; 3 Traumatology and Orthopedics, Hospital El Naranjo, Guatemala City, GTM; 4 Emergency Medicine, Hospital El Pilar, Guatemala City, GTM; 5 Emergency Medicine, Centro Médico, Guatemala City, GTM

**Keywords:** ambulatory surgical procedures, length of stay, minimally invasive procedure, patient discharge, same-day discharge

## Abstract

Same-day discharge (SDD) following minimally invasive abdominal surgery has emerged as a paradigm shift in perioperative care, aiming to enhance patient comfort and optimize healthcare efficiency. This systematic review evaluates the safety and outcomes of SDD after minimally invasive abdominal procedures. A comprehensive literature search was conducted using PubMed, Google Scholar, and the Cochrane Library for studies published between January 2015 and December 2025. The quality of randomized controlled trials and non-randomized studies was assessed using the Cochrane Risk of Bias 2.0 tool and the ROBINS-I tool, respectively, with visual summaries generated using RobVis. A total of 12 studies were included, covering procedures such as colectomy, hysterectomy, bariatric surgery, appendectomy, and cholecystectomy. The primary outcomes assessed were 30-day readmission, postoperative complications, reoperations, mortality, and ED visits. The findings indicate that, with appropriate patient selection, structured discharge planning, and adequate follow-up, SDD is a safe and effective approach. Reported readmission rates ranged from 3% to 7%, while major complication rates were low (0.9-5%). Thirty-day mortality was rare, ranging from 0% to 1% across the included studies. Post-discharge challenges such as pain, nausea, and wound complications highlight the necessity of a structured Enhanced Recovery After Surgery-based perioperative care pathway that extends beyond discharge planning to encompass all aspects of patient management. Overall, SDD appears to be a safe and feasible strategy for carefully selected patients undergoing minimally invasive abdominal surgery without increasing the risk of adverse outcomes. Successful implementation depends on strict patient selection criteria, effective perioperative planning, and robust post-discharge follow-up systems.

## Introduction and background

Perioperative care for abdominal surgery has evolved substantially over the past decade, driven by the increasing use of minimally invasive techniques, the implementation of Enhanced Recovery After Surgery (ERAS) protocols, and a growing emphasis on value-based healthcare [1]. Same-day discharge (SDD) is a direct consequence of this gradual evolution in surgical care, which has been grounded in the widespread adoption of laparoscopic methods at the end of the 20th century [2]. Laparoscopy has established the physiological basis for reassessing inpatient recovery by minimizing surgical trauma, postoperative pain, and ileus compared with open surgery. This practice shift was further reflected and justified by the systematization of ERAS practices in the 2000s, which introduced evidence-based, multimodal strategies to accelerate functional recovery and reduce perioperative stress [3,4].

Traditionally, overnight observation of patients, including those undergoing laparoscopic or robotic-assisted surgery, was conducted to detect early complications such as bleeding, pain, and nausea [5]. However, with appropriate patient selection, early discharge after surgery may be safe and does not appear to increase adverse outcomes [6,7]. SDD represents a practice change aimed at optimizing resource utilization, reducing healthcare costs, and improving patient satisfaction [8]. The principles of ERAS, which combine multimodal analgesia, early mobilization, and alleviation of perioperative stress, make this approach feasible and help accelerate recovery [9].

Within healthcare systems, SDD can help alleviate strain on bed occupancy and reduce the risk of hospital-acquired infections. For patients, it offers the psychological and logistical benefits of recovering at home [10,11]. Additionally, the drive toward SDD protocols gained renewed momentum in the post-pandemic period, as surgical backlogs persisted and the resilience of healthcare systems became increasingly important [12]. Implementing SDD requires careful perioperative planning to ensure patient safety, optimize recovery, and maintain high-quality surgical care while supporting efficient use of healthcare resources [13].

Despite its theoretical advantages, the adoption of SDD following primary abdominal surgeries remains inconsistent across institutions and surgical procedures. This reluctance is largely due to ongoing concerns about patient safety, particularly the potential for postoperative complications such as hemorrhage or severe pain after discharge. This systematic review will analyze readmission, morbidity, reoperation, and mortality rates and will examine how patient selection criteria and post-discharge milestones are associated with the effective use of SDD. Ultimately, it aims to evaluate the safety of SDD protocols following minimally invasive abdominal surgeries.

## Review

Methodology

Study Design

This systematic review was conducted in accordance with the Preferred Reporting Items for Systematic reviews and Meta-Analyses (PRISMA) 2020 guidelines. It aimed to evaluate the outcomes and safety of SDD in patients who underwent minimally invasive major abdominal surgeries.

Population, Intervention/Exposure, Comparison, and Outcomes (PICO) Framework

The research question was developed using the PICO framework, as outlined in Table 1.

**Table 1:** PICO framework SDD, same-day discharge

PICO framework	Description
Population	Adults (≥18 years) undergoing minimally invasive abdominal surgeries, including laparoscopic colectomy, total laparoscopic hysterectomy, laparoscopic cholecystectomy, and sleeve gastrectomy
Intervention/exposure	SDD: discharge within 24 hours or on the same day
Comparison	None
Outcomes	30-day readmission, 30-day overall and major complications, 30-day mortality after discharge, and ED visits after discharge

Research Question

Does SDD improve safety and surgical outcomes in adults undergoing minimally invasive abdominal surgery?

Information Sources and Search Strategy

Keywords and MeSH terms related to the PICO framework were searched using Boolean operators (AND, OR) across three electronic databases: PubMed, Cochrane Library, and Google Scholar. Only studies published in English between January 1, 2015, and December 31, 2025, were included during screening. Additionally, manual screening of reference lists from all included articles and relevant reviews was performed to identify any studies that may have been missed during the electronic search. The full search string used for each database is provided in Table 2.

**Table 2:** Search string

Database	Search string
PubMed	(("same-day discharge"[Title/Abstract] OR "same day discharge"[Title/Abstract] OR "ambulatory surgery"[Title/Abstract] OR "outpatient surgery"[Title/Abstract]) AND (Minimally Invasive Surgical Procedures [MeSH] OR minimally invasive [Title/Abstract] OR laparoscopic[Title/Abstract] OR robotic [Title/Abstract]) AND (Abdominal Surgical Procedures[MeSH]) AND(readmission[Title/Abstract] OR complications[Title/Abstract] OR mortality[Title/Abstract] OR reoperation[Title/Abstract] OR "emergency department"[Title/Abstract]))
Google Scholar	"same-day discharge" AND "minimally invasive" AND (laparoscopic OR robotic) AND ("abdominal surgery" OR "abdominal procedure") AND ("30-day readmission" OR "30-day complications" OR "major complications" OR "reoperation within 30 days" OR "30-day mortality" OR "emergency department visits")
Cochrane	("same-day discharge" OR "same day discharge" OR "ambulatory surgery" OR "outpatient surgery" OR "day-case surgery") AND ("minimally invasive surgery" OR laparoscopic OR "laparoscopic surgery" OR robotic OR "robotic surgery") AND (abdominal OR abdomen OR "abdominal surgery" OR "abdominal procedures") AND (readmission OR "hospital readmission" OR complications OR "postoperative complications" OR mortality OR death OR reoperation OR "ED visits")

Data Selection (Inclusion and Exclusion)

Randomized controlled trials (RCTs) and prospective or retrospective cohort studies were included. Studies enrolling adults (≥18 years) who underwent abdominal surgeries and were discharged on the same day of surgery or within 24 hours postoperatively were considered. Only studies written in English and published between January 1, 2015, and December 31, 2025, from peer-reviewed journals with full text available were included.

Studies were excluded if they involved non-adults or animals, were case reports, case series, abstracts, editorials, letters, reviews, or meta-analyses, lacked clear SDD timing, did not report 30-day postoperative outcomes, or had incomplete analyses.

Study Selection Process

All retrieved records were initially screened independently by two reviewers based on titles and abstracts. Full-text articles were then assessed independently by the same reviewers for eligibility. Discrepancies were resolved through discussion or, when necessary, consultation with a third reviewer to reach consensus. Reasons for full-text exclusions were recorded and are presented in a PRISMA-aligned table.

Methodological Quality Assessment

RCTs included in this review were evaluated using the Cochrane Risk of Bias (RoB) 2.0 tool, and non-RCTs were assessed using ROBINS-I. Domains were classified as low risk, some concern, or high risk of bias. Independent reviewers assessed the risk of bias, and disagreements were resolved through discussion or, if needed, consultation with a third reviewer. The evaluation was conducted transparently, with consistency and focus on the research question. To enhance clarity and provide visual interpretation, risk-of-bias traffic lights and summary plots were generated using the RobVis tool.

Data Collection and Items

Data were collected using a standardized Excel spreadsheet (Microsoft Corporation, Redmond, WA, USA). Items included study ID (first author and year), country, center type (single/multi), study design (RCT/prospective/retrospective), surgery type and approach (minimally invasive or robotic), total sample size (N), planned SDD (N), actual SDD (N, if different), criteria for SDD (eligibility/discharge criteria), follow-up duration and completeness, 30-day readmission (events/N), 30-day overall complications (events/N), 30-day major complications (Clavien ≥III; events/N), reoperations within 30 days (events/N), 30-day mortality (events/N), and ED visits at seven and 30 days (events/N). Data from all 12 included studies were extracted and presented in tabular form in the Results section.

Data Synthesis

The narrative synthesis was conducted in a structured and transparent manner to ensure rigor. (1) Initial coding involved two reviewers independently coding both quantitative and qualitative outcome data into preliminary outcome categories. (2) Theme development used these initial codes to identify broad thematic domains, which guided the organization of the evidence. The findings were then presented narratively and supported with tables to facilitate comparison between studies and highlight common trends. Heterogeneous outcome measures were systematically integrated using this thematic synthesis approach. A meta-analysis was not performed due to the heterogeneity of the data and reported outcomes.

Results

Selection Process of Studies

**Figure 1:**
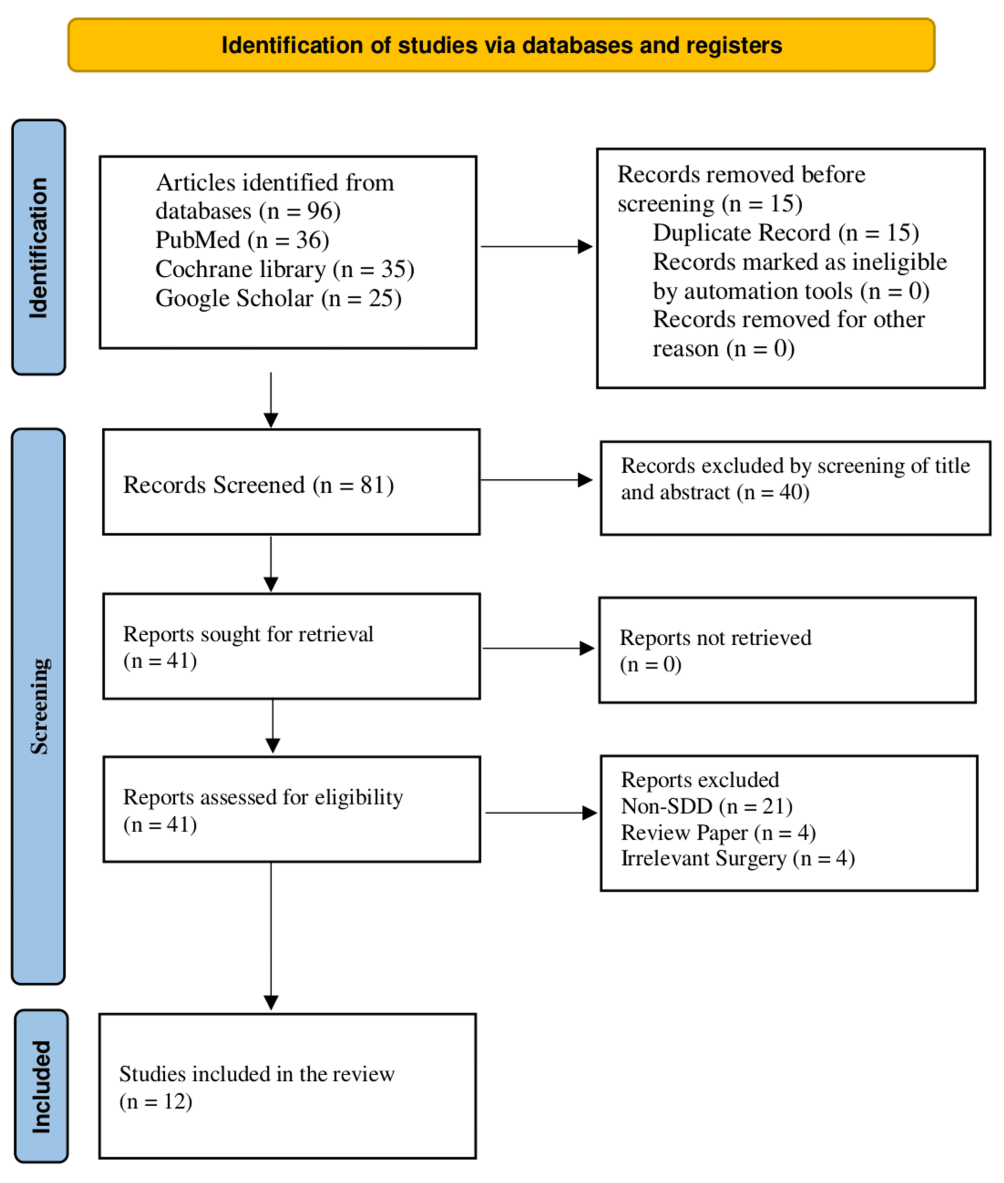
PRISMA flowchart PRISMA, Preferred Reporting Items for Systematic reviews and Meta-Analyses

A total of 96 records were identified through database searches (PubMed = 36, Cochrane Library = 35, and Google Scholar = 25). After removing 15 duplicates, 81 records remained and were screened based on titles and abstracts, resulting in the exclusion of 40 irrelevant articles. The remaining 41 articles underwent full-text review for eligibility. Of these, 29 were excluded: 21 did not involve SDD (non-SDD), four were review-based studies, and four involved irrelevant surgeries. Ultimately, 12 studies were included in the systematic review (Figure 1).

Characteristics of Included Studies

The evidence indicates that SDD is feasible across various surgical interventions, including colectomy, hysterectomy, bariatric surgery, appendectomy, and cholecystectomy. Implementation success rates are generally high, with some studies reporting actual SDD rates exceeding 90% of targeted cases when strict eligibility criteria are met. These criteria typically included American Society of Anesthesiologists (ASA) I-II status, adequate home support, and achievement of specific postoperative milestones. The high success rate of SDD reflects careful patient selection, structured perioperative management, and the importance of patient literacy and adherence to postoperative instructions.

Overall, the rate of significant safety events following SDD is low. Within 30 days, readmission rates among SDD patients ranged from 0% to 7%, with most studies reporting 2-5%. Notably, there was no statistically significant increase in 30-day mortality (which was 0% across these trials) or major complications (Clavien-Dindo III) in SDD patients. Risk ratios for severe complications, including bleeding, were not significantly different between SDD and inpatient groups in bariatric surgery studies.

Secondary outcomes indicate that severe morbidity is rare, while minor postoperative issues are more common. Reoperation rates within 30 days were generally low, ranging from 0% to 2.4%. One notable observation was that ED visits reached up to 9.2% in one bariatric study, primarily due to pain, nausea, and vomiting. Although SDD does not appear to increase major adverse events, its feasibility depends on an uncomplicated intraoperative course and stable immediate postoperative recovery, as some aspects of postoperative care are effectively transferred to the outpatient setting. Minor complications, including surgical site infections (SSIs) and postoperative nausea and vomiting (PONV), were the most frequent. Collectively, these findings indicate low rates of critical outcome events, supporting SDD as a safe and effective approach for carefully selected patients undergoing minimally invasive abdominal surgery (Table 3).

**Table 3:** Characteristics of included studies ASA, American Society of Anesthesiologists; CKD, chronic kidney disease; DVT, deep vein thrombosis; FPS-R, Faces Pain Scale, Revised; LA-C, laparoscopic appendectomy, conventional group; LA-E, laparoscopic appendectomy, early group; MBSAQIP, Metabolic and Bariatric Surgery Accreditation and Quality Improvement Program; NRS, Numeric Rating Scale; OAGB, one anastomosis gastric bypass; PACU, post-anesthesia care unit; POD, postoperative day; PONV, postoperative nausea and vomiting; RCT, randomized controlled trial; RYGB, Roux-en-Y gastric bypass; SDD, same-day discharge; SADI-S, single anastomosis duodenal-ileal bypass with sleeve gastrectomy; SG, sleeve gastrectomy; SSI, surgical site infection; UGI, upper gastrointestinal; VAS, Visual Analog Scale; VTE, venous thromboembolism

Study	Country and center type	Study design	Surgery type(s) and approach	N (total)	N (planned SDD)	N (actual SDD)	Criteria for SDD (discharge criteria)	Follow-up duration and completeness	30-day readmission: events/N	30-day overall complications: events/N	30-day major complications (Clavien III): events/N	Reoperation within 30 days: events/N	30-day mortality: events/N	ED visits seven days and 30 days: events/N
Aillaud-De-Uriarte et al. (2024) [14]	USA; single center	Retrospective cohort study	Minimally invasive colectomy	86	Not reported	41	Without significant comorbidities, mobile, tolerating oral intake, urinating, and with controlled pain/nausea	Phone call the next morning, clinic visit at two weeks	SDD: 0/41 (0%), non-SDD: 3/45 (6.7%), overall: 3/86 (3.5%)	No complications reported	Not reported	Not reported	Not reported	Not reported
Dedden et al. (2024) [15]	Netherlands; multicenter	RCT	Total laparoscopic hysterectomy	205	105	96	ASA I-II, age 18-65, home support, surgery ends before 2 PM, residence within one hour, no complex conditions	Six weeks; high follow-up	5/105 (4.8%) SDD	13/105 (12.4%) SDD (within six weeks)	1/105 (0.9%) Clavien-Dindo III SDD	Not reported	Not reported	Not reported
Cooper et al. (2024) [16]	USA; single center	Retrospective cohort study	Laparoscopic or robotic-assisted SG and RYGB	1,224	Not reported	SG: 870, RYGB: 70, total: 940	BMI ≤60, no home O₂, no CKD stage 5, low VTE risk; postoperative milestones (pain control, ambulation, oral intake, stable vitals, and voiding)	30-day follow-up	SG: 18/870 (2.1%) SDD, 11/238 (4.6%) POD1; RYGB: 0/70 SDD, 6/46 (13.1%) POD1	Not reported	Not reported	SG: 2/870 (0.2%) SDD, 1/238 (0.4%) POD1; RYGB: 0	Reported as 0% for all groups	SG: 80/870 (9.2%) SDD, 27/238 (11.3%) POD1; RYGB: 6/70 (8.6%) SDD, 12/46 (26%) POD1
Trejo-Ávila et al. (2019) [17]	Mexico; single center	RCT	Laparoscopic appendectomy	108	50 (LA-E), 58 (LA-C)	45	Ability to take oral feeding, full consciousness, ambulation alone, pain VAS <2, hemodynamic stability, able to urinate, no nausea/vomiting	30 days; complete (no loss to follow-up)	LA-E: 2/50 (4%), LA-C: 3/58 (5.2%)	LA-E: 2/50 (4%), LA-C: 3/58 (5.2%)	Not reported	LA-E: 2/50 (4%), LA-C: 3/58 (5.2%)	Not reported (likely 0)	Not reported
Mattila et al. (2016) [18]	Finland; single center	RCT	Laparoscopic cholecystectomy	167	All patients planned for day-care SDD	146	Pain/nausea controlled (NRS ≤4), mobile, tolerating oral intake, able to urinate	30-day follow-up	Ultrasonic: 3/88 (3.4%), diathermy: 4/79 (5.1%), overall: 7/167 (4.2%)	Overall: 7/167 (4.2%); events: port-site infection (4), port-site hematoma (1), DVT (1), bile leak from cystic stump (1)	Bile leak in diathermy group (1/79, 1.3%)	Bile leak	0/167 (0%)	Not reported
Gee et al. (2018) [19]	USA; single center (Children’s Health, Dallas, TX)	Prospective cohort study	Laparoscopic appendectomy	1,321	382	382/849	Patients undergoing LA for uncomplicated appendicitis. Discharge: “same calendar day” from PACU (36%) or inpatient floor (64%)	Two-week postoperative phone call (50% response rate)	2/382 (0.5%) Events: 1 intractable pain, 1 intra-abdominal abscess	SSI (superficial): 4; nausea/vomiting: 10; pain control issues: 33 (9 presented to ED)	Intra-abdominal abscess leading to readmission (1 event, likely Clavien ≥ III)	Not reported	0/382 (0%)	Breakdown: pain (9), nausea/vomiting (10), SSI (2), other (1)
Badaoui et al. (2016) [20]	France; single center	Prospective observational cohort	Laparoscopic SG	100	100	92/100 (92%)	Validated using the Aldrete score after PACU. Resumption of oral intake, no clinical/biological/radiological anomalies on routine UGI study	Phone calls on the day of surgery and POD1, clinic visits POD4, one and three months	7/100 (7%); causes: gastric leak (3), gastric stricture (1), pneumonia (1), hematoma (1), abdominal pain from splenic ischemia (1)	Events in seven patients; plus unscheduled consultations (not leading to admission): seven events in six patients (abdominal pain, dysphagia, and wound issues)	Major complications (Clavien III): 5/100 (5%) (gastric fistula 3, postoperative hematoma 1, gastric stricture 1)	3/100 (3%) (for gastric fistula)	0/100 (0%)	Not reported
Dewinter et al. (2016) [21]	Belgium; single center	Double-blind, randomized, placebo-controlled trial	Laparoscopic sterilization (day-case surgery)	79 (analyzed from 80)	All planned for day-case SDD	Implied 100% for eligible day-case patients	NRS pain ≤3, stable vital signs, ability to ambulate, absence of urinary retention	24-hour telephone interview post-discharge	Reported complications (within 24 hours): postoperative nausea (NRS >0) higher in lidocaine group; PONV requiring rescue medication: Placebo 1/40 vs. Lidocaine 7/39	Not reported	Not reported	Not reported	Not reported	Not reported
Kaushal-Deep et al. (2019) [22]	India; government tertiary teaching hospital	Prospective randomized triple-blind study	Laparoscopic cholecystectomy (4-port)	191	Not explicitly stated	106	VAS ≤3, NRS ≤3, FPS-R ≤2; no rescue analgesia; ambulated ≥1; passed urine; tolerating oral sips	Clinic visits at one week, six weeks, six months; telephone contact available	Not reported	Only two patients returned within hours for abdominal pain (managed conservatively)	Not reported	Not reported	Not reported	2/191 (within 72 hours)
Dreifuss et al. (2022) [23]	USA; multicenter (MBSAQIP)	Retrospective registry analysis	Laparoscopic SG	466,270	Not reported	14,624	Based on clinical judgment and stable postoperative status	30-day follow-up via the MBSAQIP database	SDD: 431/14,624 (2.9%), inpatient: 13,546/451,646 (3%)	Nausea/vomiting 36.6%, abdominal pain 10.2%	SDD: 0.7% (~102/14,624)	SDD: 0.1% (~15/14,624)	Not reported	Not reported
Deffain et al. (2024) [24]	Canada; single center	Retrospective cohort study	SADI-S, RYGB, OAGB, SAS I (laparoscopic or robotic)	208	208 (all planned SDD)	199 (95.7%)	Discharge if modified PACU criteria met	Telephone call on day 1, clinic visit within the first week if needed, then at one, six, and 12 months	12/208 (5.8%)	Overall morbidity: 31/208 (14.9%)	Major complications (Clavien III): 8/208 (3.9%)	5/208 (2.4%)	0/208 (0%)	9/208 (4.3%)
Aryaie et al. (2021) [25]	USA and Canada; multicenter (MBSAQIP registry)	Retrospective cohort study	Laparoscopic SG	271,658	Not reported	7,825	Not reported	30 days; completeness implied by MBSAQIP registry follow-up protocol	SDD: 262/7,813 (3.35%), inpatient (POD1-4): 218/7,813 (2.79%), RR 1.202 (1.009-1.432), p = 0.039	SDD: 90/7,813 (1.15%), Inpatient: 79/7,813 (1.01%), RR 1.139 (0.842-1.541), p = 0.397	Clavien-Dindo: leak (≥IIIb) SDD: 44/7,813 (0.56%), inpatient: 31/7,813 (0.40%), p = 0.13; bleeding (≥IIIa/IIIb) SDD: 30/7,813 (0.38%), inpatient: 24/7,813 (0.31%), p = 0.41	SDD: 63/7,813 (0.81%), inpatient: 44/7,813 (0.56%), RR 1.432 (0.975-2.104), p = 0.066	SDD: 0/7,813, inpatient: 1/7,813 (0.01%), p = 0.317	SDD: 318/7,813, inpatient: 441/7,813

Reporting Risk of Bias

**Figure 2:**
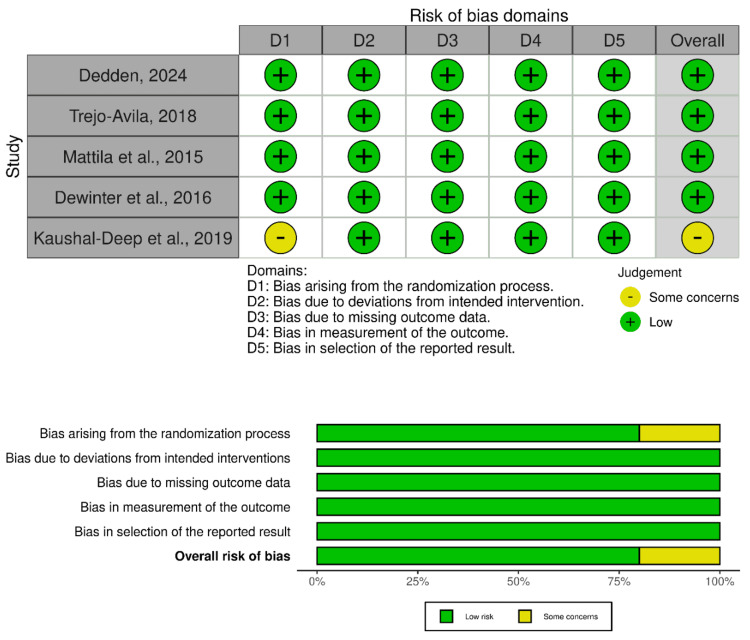
Cochrane RoB 2.0 assessment of included RCTs RCT, randomized controlled trial; RoB, Risk of Bias [15,17,18,21,22]

Five of the 12 included studies were RCTs. Four of the five RCTs exhibited low risk of bias, while one study showed some concerns or unclear risk of bias. Overall, the evidence from RCTs is considered low risk of bias (Figure 2).

**Figure 3:**
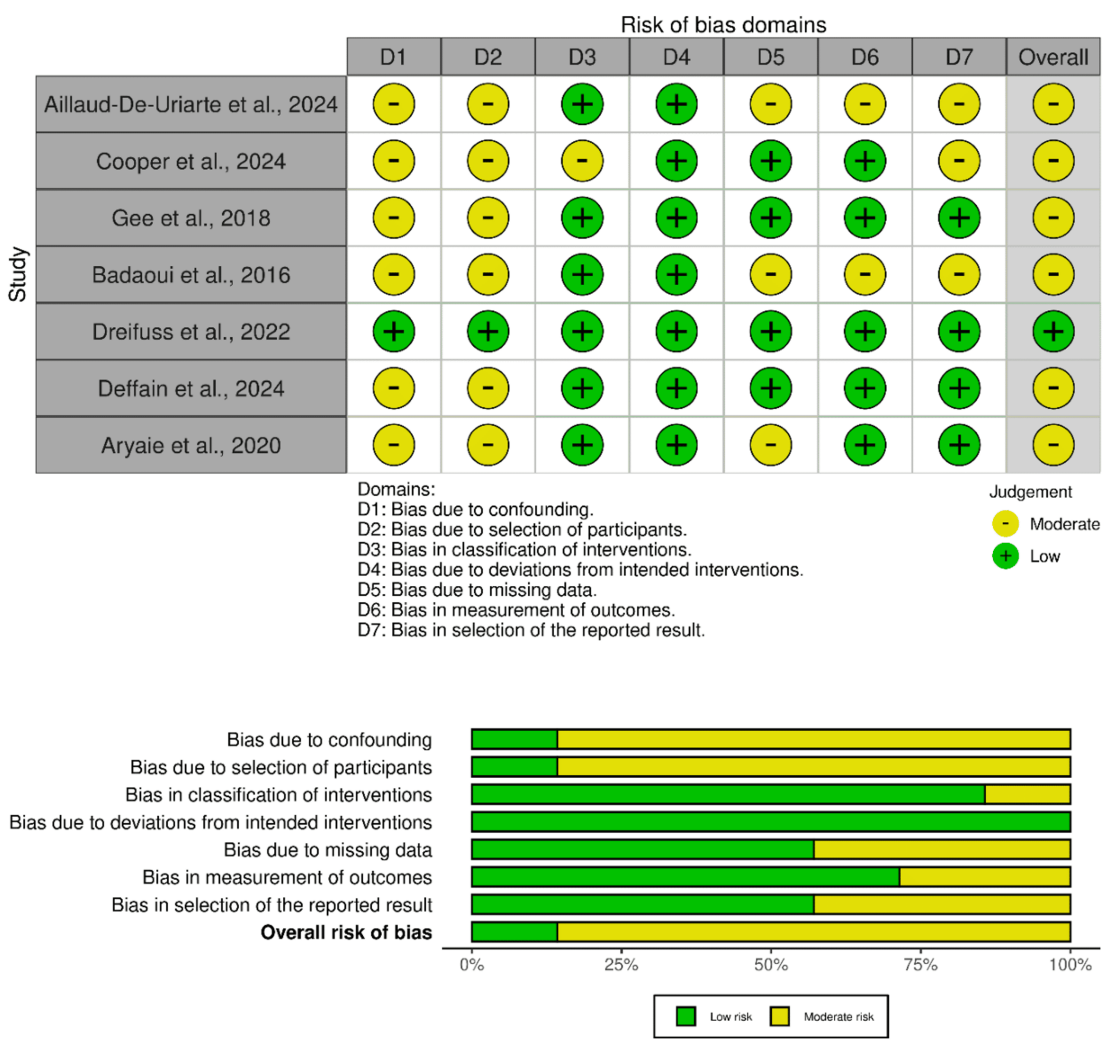
ROBINS-I quality assessment of included non-RCTs RCT, randomized controlled trial [14,16,19,20,23-25]

Of the 12 studies, seven were non-RCTs. Six of these exhibited a moderate risk of bias, and one study demonstrated a low risk of bias. Overall, the evidence from non-RCTs is considered moderate to low risk of bias. The overall quality of evidence is downgraded to moderate risk of bias due to issues in the D1 and D2 domains (Figure 3).

Discussion

30-Day Readmission Rate

The 30-day readmission rate is an important indicator for assessing the safety of SDD protocols, as it reflects whether patients required urgent hospital care shortly after returning home. Readmissions may indicate early postoperative complications or inadequate recovery at home. This review found that readmission rates were generally low across studies, supporting the feasibility of SDD. Aryaie et al. (2021) reported a readmission rate of 3.35% among SDD patients who underwent laparoscopic sleeve gastrectomy, which was slightly higher than that observed in inpatients [25]. Similarly, Dedden et al. (2024) reported a 4.8% readmission rate in their SDD group following total laparoscopic hysterectomy [15]. These findings suggest that SDD is not a major contributor to readmission risk when patients are appropriately selected and receive adequate postoperative support. Nonetheless, readmission rates vary depending on the type of surgery and patient demographics, highlighting the importance of individualized discharge planning. Overall, the literature indicates that with a robust follow-up system, SDD can be implemented without a substantial increase in hospital readmissions.

30-Day Overall Complications

Thirty-day mortality is a widely recognized objective measure of surgical safety. Among patients selected for SDD, 30-day mortality was extremely low, with several studies reporting no deaths. For instance, Aryaie et al. (2021) observed zero deaths among 7,813 SDD sleeve gastrectomy patients, compared to one death in the inpatient group [25]. Similarly, Badaoui et al. (2016) and Mattila et al. (2016) reported no mortality in their SDD cohorts [18,20]. It is important to note, however, that most of these studies involved low-risk patients (ASA grade I-II) undergoing relatively minor or intermediate procedures, in which perioperative mortality is expected to be minimal. Therefore, while these findings suggest that SDD can be safely implemented in carefully selected low-risk patients, caution is warranted when generalizing to higher-risk populations or more complex surgeries. Continuous monitoring and strict patient selection remain essential when expanding SDD protocols.

30-Day Major Complications (Clavien-Dindo ≥ III)

Major complications, defined as Clavien-Dindo grade III or higher, are events that require surgical, endoscopic, or radiological intervention or result in organ dysfunction or ICU admission. This measure is a critical indicator of surgical safety. Across studies, the incidence of major complications among SDD patients was very low. Leaks and bleeding were the most serious complications reported by Aryaie et al. (2021), occurring in 0.56% of patients undergoing SDD sleeve gastrectomy, a rate not significantly different from inpatients [25]. In their outpatient sleeve gastrectomy cohort, Badaoui et al. (2016) reported a 5% incidence of major complications, including gastric fistula and hematoma requiring surgery [20]. Mattila et al. (2016) observed a 1.3% rate of bile leaks in day-case cholecystectomy patients [18]. These findings indicate that while major complications are severe, their occurrence is infrequent. They also underscore the importance of thorough preoperative evaluation and meticulous intraoperative technique to minimize risks. Overall, SDD appears to be safe in terms of significant morbidity when applied according to accepted clinical guidelines.

Reoperation Rate Within 30 Days

The 30-day reoperation rate reflects the need for unexpected surgical intervention after the initial procedure, typically due to complications such as bleeding, anastomotic leak, or infection. Reoperation rates among SDD patients were generally low. Aryaie et al. (2021) reported a reoperation rate of 0.81% for SDD sleeve gastrectomy patients compared to 0.56% for inpatients [25]. Badaoui et al. (2016) found a 3% reoperation rate in outpatient sleeve gastrectomy, primarily related to gastric fistula [20]. Trejo-Ávila et al. (2019) reported a 4% reoperation rate among SDD appendectomy patients [17]. These findings indicate that reoperations are required in only a small proportion of SDD cases and are not significantly more frequent than inpatients. This supports the conclusion that SDD does not compromise the management of severe complications, provided that patients can be readmitted promptly if necessary. Patient education remains essential to ensure that warning signs are recognized and addressed without delay.

30-Day Mortality

The most objective safety outcome is 30-day mortality, defined as death within one month of surgery. Among SDD patients, 30-day mortality was extremely low, with many studies reporting zero deaths. Aryaie et al. (2021) observed no deaths among 7,813 SDD sleeve gastrectomy patients, compared to one death in the inpatient group [25]. Similarly, Badaoui et al. (2016) and Mattila et al. (2016) reported zero mortality in their SDD cohorts [18,20]. These findings indicate that SDD does not increase mortality risk for patients undergoing minimally invasive abdominal surgery. While mortality remains a concern for all major surgeries, these results suggest that SDD can be safely implemented without compromising patient survival. Continuous monitoring is essential, especially when expanding SDD protocols to larger patient populations.

ED Visits Within Seven to 30 Days

ED visits within the first month after discharge typically result from uncontrolled pain, nausea, wound complications, or other issues requiring unscheduled care. This outcome reflects both patient distress and the burden on emergency services. Ensuring informed patient consent prior to discharge is essential to support autonomy and prevent coercion. Cooper et al. (2024) reported a 9.2% ED visit rate among SDD sleeve gastrectomy patients, while Aryaie et al. (2021) reported 4.1% [16,25]. Similarly, Gee et al. (2018) noted that frequent ED visits after SDD appendectomy in children were primarily due to pain and nausea [19]. Although these visits do not always result in readmission, they indicate gaps in postoperative management or patient preparation. High ED utilization can undermine the efficiency of SDD and increase healthcare costs. Optimizing discharge guidelines, providing adequate analgesia, and ensuring accessible follow-up are critical for minimizing unnecessary ED visits. Proactive telephone follow-up and effective contingency plans have been shown to reduce these occurrences.

Pain Control Issues

Effective postoperative pain management is essential for patient satisfaction, mobility, and safe home recovery. Inadequate analgesia can lead to ED visits, readmission, or persistent pain. Gee et al. (2018) found that pain was the most frequent cause of ED visits following SDD appendectomy, occurring in 9% of their pediatric cohort [19]. Similarly, Kaushal-Deep et al. (2019) used a pain score of ≤3 as a discharge criterion for cholecystectomy patients [22]. While most SDD protocols include planned pain management, some patients continue to experience significant pain at home. This underscores the need for multimodal analgesia and patient education regarding medication use and available support for pain crises. The success and acceptability of SDD programs depend heavily on effective pain control.

Nausea/Vomiting (PONV)

PONV are common and uncomfortable complications that can delay discharge, cause dehydration, and lead to unplanned healthcare use. Prevention of PONV is a key component of ERAS guidelines. Gee et al. (2018) reported that 10% of SDD appendectomy patients visited the ED due to nausea or vomiting [19]. Dewinter et al. (2016) highlighted that PONV not only affects patient comfort but also impedes oral intake and ambulation, which are critical milestones for SDD [21]. Standard prophylactic measures, including antiemetics, fluid management, and avoidance of triggering anesthetics, are used to reduce PONV. Although PONV occurs in a minority of SDD patients despite these measures, patients with significant nausea or vomiting before discharge should remain under observation.

SSI

SSI is a common healthcare-associated infection that can lead to wound dehiscence, sepsis, readmission, and additional surgery. In SDD settings, SSI rates can be monitored to assess infection control. Gee et al. (2018) reported a low incidence of superficial SSI in SDD appendectomy patients, and Mattila et al. (2016) noted port-site infections following cholecystectomy [18,19]. The occurrence of SSI highlights the importance of preoperative skin preparation, sterile technique, and postoperative wound care education. Early discharge necessitates that patients are trained to identify signs of infection. Low reported SSI rates suggest that SDD is not inherently associated with increased infection risk, likely due to the minimally invasive nature of the procedures and reduced hospital exposure. Nevertheless, close follow-up is essential to detect and manage SSIs promptly.

Patient Selection Criteria for SDD

The safety and effectiveness of SDD programs rely on careful patient selection. Common criteria include ASA physical status I-II, age limits, absence of significant comorbidities, adequate home support, proximity to the hospital, and psychological readiness. Dedden et al. (2024) selected ASA I-II patients aged 18-65 with home support for hysterectomy [15]. Cooper et al. (2024) excluded patients with high BMI, home oxygen requirements, or severe chronic kidney disease in bariatric SDD [16]. These criteria help identify low-risk patients suitable for home recovery. Differences in guidelines across surgical procedures underscore the need for procedure- and patient-specific protocols. Appropriate patient selection reduces complications and readmissions, contributing to the generally positive outcomes observed in the reviewed studies. As SDD is applied to more complex cases, refinement of selection criteria will remain essential.

Discharge Readiness Milestones

Discharge readiness milestones are objective clinical criteria that a patient must meet to be safely discharged on the same day. Common milestones include adequate pain control, the ability to ambulate independently, tolerance of oral intake, spontaneous urination, and stable vital signs. Trejo-Ávila et al. (2019) required full consciousness, independent ambulation, pain Visual Analog Scale <2, and absence of nausea for appendectomy patients [17]. Kaushal-Deep et al. (2019) applied similar benchmarks for cholecystectomy patients [22]. Achieving these milestones ensures that patients have sufficiently recovered from anesthesia and are capable of performing basic self-care at home. Consistency in SDD protocols is enhanced by the systematic use of standardized discharge criteria, which improves safety. Evidence indicates that adherence to these milestones is associated with lower complication and readmission rates, supporting their clinical relevance.

Follow-Up Completeness

Follow-up completeness refers to the proportion of patients who participate in planned postoperative evaluations through phone calls, clinic visits, or electronic surveys. Follow-up rates varied across studies. For example, Gee et al. (2018) achieved a 50% response rate for postoperative phone calls, potentially underestimating complications [19], whereas Trejo-Ávila et al. (2019) had 100% follow-up at 30 days with no loss to follow-up [17]. High follow-up completeness is essential for accurate safety monitoring, as it allows reliable assessment of post-discharge events. Effective follow-up requires allocation of resources, patient engagement, and, in some cases, utilization of electronic health platforms.

Procedure-Specific Complications

Procedure-specific complications are unique to certain surgeries, such as bile leaks after cholecystectomy, gastric leaks after bariatric surgery, or intra-abdominal abscesses after appendectomy. Monitoring these complications is critical for evaluating SDD safety in each procedure. Mattila et al. (2016) reported a 1.3% incidence of bile leaks in day-case cholecystectomy patients [18]. Badaoui et al. (2016) found that 3% of outpatient sleeve gastrectomy patients experienced a gastric fistula [20]. A few cases of intra-abdominal abscess occurred in SDD appendectomy patients [19]. Although these complications can be severe, their incidence in SDD cohorts does not exceed expected rates. Thus, SDD can be safely applied even for procedures with known specific risks, provided patient selection and surgical technique are appropriate.

Length of Follow-Up Duration

Follow-up duration varied among studies, ranging from short-term (e.g., 24 hours) to several months. Short-term follow-up may miss delayed complications, whereas longer follow-up (e.g., 30 days or six weeks) provides a more complete safety profile. Dewinter et al. (2016) conducted a 24-hour follow-up after laparoscopic sterilization, focusing on immediate recovery [21]. In contrast, Dedden et al. (2024) followed patients for up to six weeks, and Deffain et al. (2024) extended follow-up to 12 months [15,24]. Follow-up duration affects reported readmission, complication, and ED visit rates. A longer follow-up is preferable to capture both early and late adverse events. Standardization of follow-up across studies would improve comparability and strengthen evidence for SDD protocols.

Strengths and Clinical Implications

The primary strength of this evidence lies in its methodological diversity and external validity. RCTs provide high-quality causal evidence, while large-scale retrospective cohort studies using national registries (e.g., Metabolic and Bariatric Surgery Accreditation and Quality Improvement Program) offer generalizability and statistical power to detect rare events such as mortality. This combination supports the conclusion that SDD is feasible and safe in appropriately selected patients.

Clinically, these findings promote a paradigm shift in postoperative care. SDD protocols do not increase risk in ASA I-II patients who meet postoperative milestones, including controlled pain, ambulation, and oral intake. Implementing SDD can optimize healthcare resource use, reduce inpatient bed pressure, and lower costs. Experience across bariatric, colorectal, gynecologic, and general surgery demonstrates the model’s broad applicability in minimally invasive procedures.

Limitations and Future Recommendations

Several limitations affect the generalizability of findings. Selection bias is a concern because SDD patients are typically healthier and lower risk, which can complicate comparisons with inpatient groups. Outcomes were inconsistently measured, and follow-up was sometimes insufficient, risking underestimation of adverse events. Important patient-centered outcomes, such as satisfaction and quality of life, were often not assessed. Future studies should employ pragmatic RCTs or propensity score-matched designs, adopt standardized outcome sets focusing on graded complications and patient-reported outcomes, and establish uniform follow-up protocols. Structured follow-up through phone or video calls at six, 12, and 24 hours post-discharge is recommended to enhance patient safety, support recovery, and improve healthcare efficiency.

## Conclusions

SDD can be a safe and effective approach for appropriately selected patients without consistently increasing complications or readmissions. Its success relies on careful patient selection, clear discharge criteria, and structured follow-up support. When implemented correctly, SDD can improve healthcare efficiency while maintaining high safety standards.

## References

[1] McLemore EC, Lee L, Hedrick TL, Rashidi L, Askenasy EP, Popowich D, Sylla P (2022). Same day discharge following elective, minimally invasive, colorectal surgery: a review of enhanced recovery protocols and early outcomes by the SAGES Colorectal Surgical Committee with recommendations regarding patient selection, remote monitoring, and successful implementation. Surg Endosc.

[2] Kiran RP, Herman K, Khoshknabi D, Angistriotis A, Church JM (2022). Feasibility and safety of ambulatory surgery as the next management paradigm in colorectal resection surgery. Ann Surg.

[3] Hickman LC, Paraiso MF, Goldman HB, Propst K, Ferrando CA (2021). Same-day discharge after minimally invasive sacrocolpopexy is feasible, safe, and associated with high patient satisfaction. Female Pelvic Med Reconstr Surg.

[4] Robison EH, Smith PE, Pandya LK, Nekkanti S, Hundley AF, Hudson CO (2022). Readmissions and perioperative outcomes for same-day versus next-day discharge after prolapse surgery. Int Urogynecol J.

[5] Lee L, Eustache J, Tran-McCaslin M (2022). North American multicentre evaluation of a same-day discharge protocol for minimally invasive colorectal surgery using mHealth or telephone remote post-discharge monitoring. Surg Endosc.

[6] Pang G, Kwong M, Schlachta CM, Alkhamesi NA, Hawel JD, Elnahas AI (2021). Safety of same-day discharge in high-risk patients undergoing ambulatory general surgery. J Surg Res.

[7] Ortega PM, Sabatella L, Ahmed AR (2025). Safety outcomes in same-day discharge anastomotic metabolic/bariatric surgery vs regular overnight discharge protocol: a systematic review and meta-analysis. Obes Surg.

[8] Penner KR, Fleming ND, Barlavi L, Axtell AE, Lentz SE (2015). Same-day discharge is feasible and safe in patients undergoing minimally invasive staging for gynecologic malignancies. Am J Obstet Gynecol.

[9] Lee L, Eustache J, Baldini G (2022). Enhanced recovery 2.0 - same day discharge with mobile app follow-up after minimally invasive colorectal surgery. Ann Surg.

[10] Molina JC, Misariu AM, Nicolau I, Spicer J, Mulder D, Ferri LE, Mueller CL (2018). Same day discharge for benign laparoscopic hiatal surgery: a feasibility analysis. Surg Endosc.

[11] Applebaum JC, Kim EK, Rush M, Shah DK (2023). Safety of same-day discharge versus hospital admission in minimally invasive myomectomy. J Minim Invasive Gynecol.

[12] Sharma S, Surve A, Cottam D, Wooley A, Christensen J, Sharma S, Patel T (2025). Safety of same-day discharge bariatric surgery: a comprehensive analysis of 457 cases across multiple procedure types. Obes Surg.

[13] Landreneau JP, Strong AT, Ponsky JL, Tu C, Kroh MD, Rodriguez JH, El-Hayek K (2020). Enhanced recovery outcomes following per-oral pyloromyotomy (POP): a comparison of safety and cost with same-day discharge versus inpatient recovery. Surg Endosc.

[14] Aillaud-De-Uriarte D, Hernandez-Flores LA, Hernandez-Moreno A, Zachariah PN, Bhatia R, Rodriguez-Gaytan J, Marines-Copado D (2024). Same-day discharge after a minimally invasive colectomy: a successful approach to patient selection. Cureus.

[15] Dedden SJ, Maas JW, Smeets NA (2024). Same-day discharge after laparoscopic hysterectomy for benign/premalignant disease: a multicentre randomised controlled trial. BJOG.

[16] Cooper S, Patel S, Wynn M, Provost D, Hassan M (2024). Outcomes of same-day discharge in bariatric surgery. Surg Endosc.

[17] Trejo-Ávila ME, Romero-Loera S, Cárdenas-Lailson E, Blas-Franco M, Delano-Alonso R, Valenzuela-Salazar C, Moreno-Portillo M (2019). Enhanced recovery after surgery protocol allows ambulatory laparoscopic appendectomy in uncomplicated acute appendicitis: a prospective, randomized trial. Surg Endosc.

[18] Mattila A, Mrena J, Kautiainen H, Nevantaus J, Kellokumpu I (2016). Day-care laparoscopic cholecystectomy with diathermy hook versus fundus-first ultrasonic dissection: a randomized study. Surg Endosc.

[19] Gee K, Ngo S, Burkhalter L, Beres AL (2018). Safety and feasibility of same-day discharge for uncomplicated appendicitis: a prospective cohort study. J Pediatr Surg.

[20] Badaoui R, Alami Chentoufi Y, Hchikat A (2016). Outpatient laparoscopic sleeve gastrectomy: first 100 cases. J Clin Anesth.

[21] Dewinter GB, Teunkens A, Vermeulen K, Al Tmimi L, Van de Velde M, Rex S (2016). Systemic lidocaine fails to improve postoperative pain, but reduces time to discharge readiness in patients undergoing laparoscopic sterilization in day-case surgery: a double-blind, randomized, placebo-controlled trial. Reg Anesth Pain Med.

[22] Kaushal-Deep SM, Lodhi M, Anees A, Khan S, Khan MA (2019). Randomised prospective study of using intraoperative, intraincisional and intraperitoneal ropivacaine for the early discharge of post-laparoscopic cholecystectomy patients as a day case in a cost-effective way in government setup of low-income and middle-income countries: opening new horizons. Postgrad Med J.

[23] Dreifuss NH, Xie J, Schlottmann F (2022). Risk factors for readmission after same-day discharge sleeve gastrectomy: a metabolic and bariatric surgery accreditation and quality improvement program database analysis. Obes Surg.

[24] Deffain A, Denis R, Alfaris H (2024). Anastomotic metabolic and bariatric surgeries with same-day discharge: 30-day outcomes of a cohort from a high-volume center in Canada. Surg Obes Relat Dis.

[25] Aryaie AH, Reddy V, Dattilo Z, Janik MR (2021). Safety of same-day discharge after laparoscopic sleeve gastrectomy: propensity score-matched analysis of the Metabolic and Bariatric Surgery Accreditation and Quality Improvement Program Registry. Surg Obes Relat Dis.

